# The balanced unsaturated fatty acid supplement constituted by woody edible oils improved lipid metabolism and gut microbiota in high-fat diet mice

**DOI:** 10.3389/fnut.2023.1203932

**Published:** 2023-07-20

**Authors:** Xi Chen, Jingqi Ran, Muhammad Mazhar, Yong Zhu, Yichen Lin, Likang Qin, Song Miao

**Affiliations:** ^1^Key Laboratory of Plant Resource Conservation and Germplasm Innovation in Mountainous Region (Ministry of Education), College of Life Sciences/Institute of Agro-Bioengineering, Guizhou University, Guiyang, China; ^2^Department of Laboratory Medicine, Affiliated Jinyang Hospital of Guizhou Medical University, Guiyang, China; ^3^School of Liquor and Food Engineering, Guizhou University, Guiyang, China; ^4^Teagasc Food Research Centre, Moorepark, Fermoy, Co. Cork, Ireland

**Keywords:** woody edible oil, unsaturated fatty acid, blended oil, lipid metabolism, gut microbiota

## Abstract

The dietary intervention has demonstrated effectiveness in improving hyperlipidemia and obesity. Woody edible oils are rich in unsaturated fatty acids (UFAs) that could positively affect lipid metabolism. In this study, the blended oil (BLO), a balanced UFA supplement, constituted by *Zanthoxylum bungeanum* (Chinese Red Pepper) seed oil, walnut (*Juglans regia*) oil, camellia (*Camema oleifera*) seed oil and perilla (*Perilla frutescens*) seed oil was established referring to the Chinese dietary reference intakes, in which the ratios of monounsaturated/polyunsaturated fatty acids and ω-6/ω-3 polyunsaturated fatty acids were 1:1 and 4:1, respectively. The BLO was administrated to KM mice fed a high-fat diet (HFD) by gavage every day at a dose of 3.0 mL/kg·bw for 10 weeks to assess its effects on serum lipid levels, liver antioxidant activities and gut microbial composition. The results showed that the BLO improved hepatic steatosis, liver oxidative stress, and serum lipid levels. Additionally, there was an increased abundance of *Lactobacillus*, *Allobaculum*, and *Blautia*, along with a decreased abundance of *Staphylococcus* in cecal contents. These changes were found to be positively correlated with the metabolic improvements, as indicated by Spearman’s correlation analysis. These findings implied the practicality of the balanced unsaturated fatty acid consumption in preventing hyperlipidemia and obesity.

## Graphical abstract

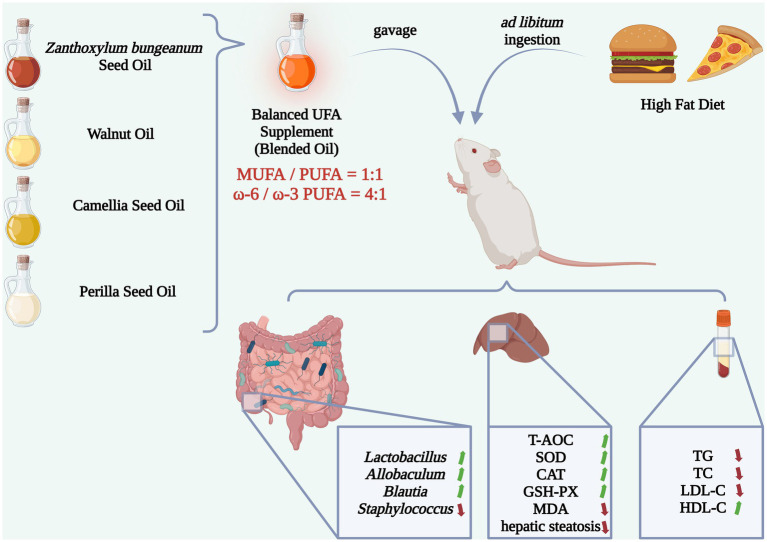

## 1. Introduction

Hyperlipidemia and obesity are significant public health crises contributing to numerous metabolic disorders (1). Dietary intervention continues to be the primary recommendation for obesity management (2). Previous studies have demonstrated a positive association between elevated intake of saturated fatty acids (SFAs) and morbidity related to specific metabolic disorders (3, 4). Thus, replacing SFAs with unsaturated fatty acids (UFAs) is recommended for improving dietary patterns (5). Essential UFAs are abundant in most edible oils, particularly vegetable oils (6). Woody oil plants, widely distributed in southern China and other regions, are essential sources of edible oil (7, 8). Woody edible oils are rich in monounsaturated fatty acids (MUFAs), ω-3 and ω-6 polyunsaturated fatty acids (PUFAs), as well as other nutrients such as antioxidant peptides, phytosterols, and polyphenols, which could reduce serum triglyceride (TAG), enhance antioxidant and antibacterial activities, and prevent various metabolic disorders (7–9).

The most frequently used cooking oils in modern diets are commonly yielded from grains and vegetables (e.g., soybean, corn, canola, and sunflower), which contain higher levels of ω-6 PUFAs than ω-3 PUFA (10). Unfortunately, the increasing consumption of processed food exacerbates this problem (11). The high ω-6/3 PUFA ratio in diets could influence lipid balance, leading to the development of specific diseases and an increased risk of death (12). Various chronic metabolic disorders, such as obesity, fatty liver disease, and hyperlipidemia, have been reported to be associated with the increased ω-6/3 PUFA ratio in diets (13). Hence, numerous studies have emphasized the importance of balanced UFAs for maintaining human health (11–14).

The rational ingestion of PUFAs is vital. Given that no single oil satisfies all the nutritional requirements, it is practical to use a blend of edible oils to produce the specific dietary product with an improved ω-6/3 PUFA ratio (15). Blended oil (BLO), now widely accepted in many countries, provides the food industry with more flexibility (16). Numerous oil blend products involving camellia seed, olive, soybean, and other unconventional oil (e.g., rice bran oil) have been reported (17–19). The World Health Organization recommends a ratio of 1:1.5:1 for SFAs/MUFAs/PUFAs, and a ratio of 5~10:1 for ω-6/ω-3 PUFAs in dietary oil consumption (20). Similarly, the Chinese Nutrition Association recommends the corresponding ratios of 1:1:1 and 4~6:1, respectively (21). The ω-6/ω-3 PUFA ratio of 5:1 (or even lower) could lead to upregulated peroxisome proliferator-activated receptor γ (PPAR-γ) expression, decreased levels of nuclear factor κB (NF-κB), aortic reactive oxygen species (ROS) and total cholesterol (TC), thereby contributing to the ameliorated lipid metabolism, inflammation, and oxidative stress (22–24).

The *Zanthoxylum bungeanum* (Chinese red pepper) seeds are industrial by-products, which could be potential woody edible oil resources rich in UFAs (25). Moreover, another study has suggested that *Zanthoxylum bungeanum* seed oil possesses specific anti-cancer activities by affecting the cell cycle and apoptosis (26). Likewise, other woody plant seeds, such as walnuts (*Juglans regia*) and camellia (*Camema oleifera*) seeds, are abundant in UFAs and bioactive components (e.g., flavonoids and tocopherols) (9, 27). These woody species are widely cultivated in Guizhou Province, southwest China. Additionally, Guizhou Province is a significant habitat for perilla (*Perilla frutescens*), whose seed oil is abundant in a-linolenic acid (ALA), a vital type of PUFA (28). Herein, we reported the BLO, a balanced UFA supplement consisting of the *Zanthoxylum bungeanum* seed oil (ZanO), walnut oil (WalO), camellia seed oil (CamO) and perilla seed oil (PerO) in the ratios of 1:1 for MUFAs/PUFAs and 4:1 for ω-6/ω-3 PUFAs, respectively. The BLO was orally subjected to the KM mice fed a high-fat diet (HFD) to investigate its influences on serum lipid levels and liver antioxidant activities. Afterward, the gut microbiota of cecal contents was explored via high-throughput sequencing, and the correlations between the gut microbiota and core host parameters were analyzed. We aim to validate the enhanced nutritional characteristics and metabolic improvement functions of the balanced UFA supplement through this study, with the prospect of providing evidence for the further development of woody oil crops, improvement of dietary oils and fats, and intervention in metabolic disorders.

## 2. Materials and methods

### 2.1. Materials and reagents

The *Zanthoxylum bungeanum* seed oil, walnut oil, camellia seed oil and perilla seed oil were supplied by Guizhou Xuande Biological Co., Ltd., the Walnut Research Institute of Guizhou Academy of Forestry, Guizhou Hengshengyuan Agricultural Development Co., Ltd., and Guizhou Academy of Agricultural Sciences, respectively. Chromatography-grade methanol was acquired from Sigma Aldrich (St. Louis, MO, United States). All remaining reagents, either chromatography or analytical grade, were obtained from Sinopharm Chemical Reagent Co., Ltd. (Shanghai, China). The mouse chow diet was provided by HFK Bioscience Co., Ltd. (Beijing, China). The purified water used in this study was purchased from Watsons water (Hong Kong, China).

### 2.2. Profiling of fatty acid compositions of various oils

The fatty acid compositions were determined as follows: the oil sample (60 mg) was dissolved in 4 mL of isooctane, and 200 μL of potassium hydroxide-methanol solution (2 mol/L) was added for methyl esterification, followed by shaking vigorously for 30 s. The solution was then left to stand still until it became clear. Next, sodium bisulfate (1 g) was added to neutralize the remaining potassium hydroxide. After all the salt was precipitated, the upper layer of the solution was taken for gas chromatography (GC) analysis.

The GC analysis was performed on an Agilent 7890A gas chromatograph equipped with an Agilent J&W DB-WAX capillary column (0.25 μm × 0.25 mm × 30 m) and a flame ionization detector. The column temperature increased from 50°C to 200°C at a rate of 25°C/min and kept at 200°C for 8 min, followed by increasing to 230°C at a rate of 3°C/min. The fatty acids in the oil samples were analyzed by comparing the retention time of peaks with standard substances, following the Area Normalization Method outlined in the National Food Safety Standard of China (GB 5009.168-2016) for the determination of fatty acids in foods.

### 2.3. Preparation of blended oil

Based on the measured results of various PUFAs and MUFAs in ZanO, WalO, CamO, and PerO, a simulation calculation was conducted using MATLAB R2020b software (MathWorks 2020b) to acquire the optimal percentage of ZanO, WalO, CamO, and PerO for oil blending, which could achieve the ratios of MUFAs/PUFAs and ω-6/ω-3 PUFAs at 1:1 and 4:1, respectively. The codes and computational results are listed in Supplementary Figures 1, 2.

According to the computational results obtained using MATLAB software, ZanO, WalO, CamO, and PerO were added to the same container in the corresponding proportions (accurate to 0.01 g). After thorough mixing, the fatty acid composition of the blended oil was analyzed again using the previously described method in section 2.2.

### 2.4. Animals and diets

All the animal procedures were approved by the Laboratory Animal Ethics Committee of Guizhou University (No. EAE-GZU-2021-P006). Specific pathogen-free (SPF) Kunming (KM) mice (6-week-old, male, body weight 40.0 ± 2.0 g) were supplied from SPF (Beijing) Biotechnology Co., Ltd. (Beijing, China). The composition of the chow diet is shown in Supplementary Tables 1, 2, and the HFD were made by mixing the chow diet (fat content: 4.0%) with additional commercial materials of lard (10%), egg yolk powder (10%), cholesterol (1%) and cholate (0.2%). All the mice were acclimatized for 7 days under controlled conditions (temperature of 23°C ± 2°C, humidity of 55% ± 5%, 12 h light/dark cycle) with *ad libitum* access to a chow diet and purified water. Subsequently, the mice were randomly assigned to 12 diet-based groups (each *n* = 10). All the diets were stored at −20°C and replaced daily, as well as purified water. All the mice had *ad libitum* access to purified water and indicated diets (shown in Table 1), and the gavage was conducted every day between 9 and 11 a.m. with a dose of 3.0 mL/kg·bw per mouse for 10 weeks. Each mouse’s health condition was monitored daily, and the body weight (BW) was monitored once a week.

**Table 1:** Treatment of each group.

Group	Diet	Gavage
NC	Chow diet	Sterile water, 3.0 mL/kg·bw/day
HC	HFD	Sterile water, 3.0 mL/kg·bw/day
Zan	Chow diet	ZanO, 3.0 mL/kg·bw/day
HZan	HFD	ZanO, 3.0 mL/kg·bw/day
Wal	Chow diet	WalO, 3.0 mL/kg·bw/day
HWal	HFD	WalO, 3.0 mL/kg·bw/day
Cam	Chow diet	CamO, 3.0 mL/kg·bw/day
HCam	HFD	CamO, 3.0 mL/kg·bw/day
Per	Chow diet	PerO, 3.0 mL/kg·bw/day
HPer	HFD	PerO, 3.0 mL/kg·bw/day
Ble	Chow diet	BLO, 3.0 mL/kg·bw/day
HBle	HFD	BLO, 3.0 mL/kg·bw/day

ZanO, *Zanthoxylum bungeanum* seed oil; WalO, walnut oil; CamO, camellia seed oil; PerO, perilla seed oil; BLO, blended oil; HFD, high-fat diet.

Eventually, all overnight-fasted mice were anesthetized with diethyl ether prior to sacrifice. Serum samples were prepared by centrifuging whole blood samples collected from the orbital vein of anesthetized mice at 3,500 rpm for 15 min at 4°C. The resulting serum was stored at −80°C for further analysis. Liver, spleen, epididymal fat pad, and cecal contents (scraped under sterile conditions) were collected by dissection. A portion of the liver from each mouse was immediately separated and immersed in a 4% paraformaldehyde solution for fixation and future staining. The cecal contents and residual liver tissues were snap-frozen in liquid nitrogen and stored at −80°C for future analysis.

### 2.5. Histological analysis of liver

The fixed liver tissues were subjected to the procedures of gradient dehydration, paraffin wax embedding, sectioning (5 μm thickness), and H&E and Oil Red O staining (Wuhan Servicebio Technology Co., Ltd., Wuhan, China), according to a previous study (29). The processed liver tissues were examined with an optical microscope (Ningbo Sunny Instruments Co., Ltd., Zhejiang, China).

### 2.6. Profiling of biochemical indicators in serum and liver

The levels of total triglyceride (TG), total cholesterol (TC), low-density lipoprotein cholesterol (LDL-C), and high-density lipoprotein cholesterol (HDL-C) in serum were determined as follows: the serum samples stored at −80°C were thawed at −4°C, and the processing procedures were performed according to the manufacturer’s instructions. The absorbances of the sample-reagent mixtures were measured using a Spectra Max® 190 microplate reader (Sunnyvale, CA, United States).

The levels of total antioxidant capacity (T-AOC), superoxide dismutase (SOD), catalase (CAT), glutathione peroxidase (GSH-PX), and malondialdehyde (MDA) in liver samples were determined as follows: the liver samples stored at −80°C were thawed at −4°C, rinsed with pre-cooled normal saline, and homogenized in pre-cooled normal saline under an ice bath to obtain 10% (g/L) homogenized liver samples. The subsequent processing steps were performed according to the manufacturer’s instructions. The absorbances of the sample-reagent mixtures were measured using a Spectra Max® 190 microplate reader (Sunnyvale, CA, United States) or an L5S ultraviolet–visible spectrophotometer (Shanghai INESA, Shanghai, China), according to the manufacturer’s instructions.

All the tests were conducted using commercial assay kits (Nanjing Jiancheng Biology Engineering Institute, Nanjing, China).

### 2.7. Sequencing of the gut microbiota

Total DNA was extracted from the cecal content samples (*n* = 3 mice/group) using the Magnetic Soil and Stool DNA Kit (Tiangen Biochemical Technology Co., Ltd., Beijing, China). The DNA concentration was quantified with a Qubit 3.0 Fluorometer (Invitrogen, Carlsbad, CA, United States), and then the DNA was diluted to a concentration of 1 ng/μL with ddH_2_O for PCR. The hypervariable regions of the bacterial 16S rDNA (V3–V4) were subsequently amplified with modified universal primers: 341F (5′-CCTAYGGGRBGCASCAG-3′) and 806R (5′-GGACTACNNGGGTATCTAAT-3′) (30). The PCR amplifications were processed using the Phusion® High-Fidelity PCR Master Mix with GC Buffer and Phusion® High-Fidelity DNA polymerase (New England Biolabs, Ipswich, MA, United States) in a 30-μL reaction mixture containing 15 μL of Phusion Master Mix (2×), 10 μL of template DNA, 1 μL of each primer, and ddH_2_O diluted to a final volume of 30 μL. The thermal cycling program was conducted on a Bio-Rad T100 thermal cycler (Bio-Rad, United States) with the following conditions: 3 min of pre-denaturation at 94°C, 35 cycles of 45 s for denaturation at 94°C, 60 s for annealing at 50°C and 90 s for extension at 72°C, followed by a final extension at 72°C for 10 min. Subsequently, the integrity and purity of PCR amplicons were identified by 2% agarose gel electrophoresis, and the target DNA bands were recycled using a Universal DNA Purification Kit (Tiangen Biochemical Technology Co., Ltd., Beijing, China).

The libraries were constructed using the NEBNext® Ultra II DNA Library Prep Kit (New England Biolabs, Ipswich, MA, United States) and quantified using a Qubit 3.0 Fluorometer. The qualified libraries were loaded onto the high-throughput sequencing platform NovaSeq 6000 (Illumina, San Diego, CA, United States). The bioinformatics analysis of the amplicon sequence variants was performed using the Quantitative Insights Into Microbial Ecology 2 (QIIME2) software (version 1.9.1.). Eventually, Spearman’s correlation analysis was conducted to investigate the correlation between specific gut microbiota and core host parameters.

### 2.8. Statistical analysis

All data were expressed as mean ± SEM and evaluated by SPSS statistics software (version 19.0, IBM, Chicago, IL, United States) using one-way analysis of variance (ANOVA).

## 3. Results

### 3.1. Fatty acid compositions of various oils

The fatty acid compositions were measured using GC, and the optimal percentages of different oils were obtained via simulating computation on MATLAB software. The percentages of component oils were 24.97% for ZanO, 51.93% for WalO, 23.00% for CamO, and 0.10% for PerO, respectively. The GC chromatograms are shown in Supplementary Figure 3, and the fatty acid compositions are listed in Table 2. The MUFA-rich ZanO and CamO were abundant in palmitoleic acid (53.87% ± 2.40%) and oleic acid (81.18% ± 0.06%), respectively, while the PUFA-rich WalO and PerO were abundant in linoleic acid (62.55% ± 0.13%) and α-linolenic acid (62.29% ± 0.10%), respectively. The BLO achieved ratios of 1:1 for MUFAs/PUFAs and 4:1 for ω-6/ω-3 PUFAs, respectively.

**Table 2:** Fatty acid compositions of various oils.

	ZanO	WalO	CamO	PerO	BLO
Palmitic acid (C16:0)	10.28 ± 0.05^a^	6.58 ± 0.20^d^	7.97 ± 0.05^b^	6.26 ± 0.04^e^	7.73 ± 0.03^c^
Palmitoleic acid (C16:1, ω-7)	53.87 ± 2.40^a^	ND	0.09 ± 0.01^c^	0.09 ± 0.01^c^	16.05 ± 0.04^b^
Stearic acid (C18:0)	0.63 ± 0.12^d^	2.25 ± 0.05^b^	2.48 ± 0.03^a^	2.10 ± 0.05^b^	1.89 ± 0.08^c^
Oleic acid (C18:1, ω-9)	18.19 ± 0.32^c^	15.70 ± 0.16^e^	81.18 ± 0.06^a^	16.25 ± 0.05^d^	28.75 ± 0.05^b^
Linoleic acid (C18:2, ω-6)	7.74 ± 0.78^d^	62.55 ± 0.13^a^	7.22 ± 0.05^d^	12.58 ± 0.03^c^	36.18 ± 0.03^b^
α-linolenic acid (C18:3, ω-3)	9.17 ± 1.40^c^	12.67 ± 0.08^b^	0.38 ± 0.02^d^	62.29 ± 0.10^a^	9.05 ± 0.04^c^
Arachidic acid (C20:0)	ND	ND	0.22 ± 0.19^b^	0.27 ± 0.03^a^	0.14 ± 0.01^ab^
Eicosenoic acid (C20:1, ω-9)	ND	0.21 ± 0.02^b^	0.51 ± 0.05^a^	0.17 ± 0.01^c^	0.21 ± 0.01^b^
SFA	11.02 ± 0.10^a^	8.83 ± 0.15^d^	10.67 ± 0.15^b^	8.63 ± 0.06^e^	9.76 ± 0.05^c^
MUFA	72.06 ± 2.08^b^	15.95 ± 0.10^e^	81.79 ± 0.07^a^	16.51 ± 0.04^d^	45.02 ± 0.08^c^
PUFA	16.91 ± 2.18^c^	75.22 ± 0.20^a^	7.60 ± 0.03^d^	74.86 ± 0.08^b^	45.22 ± 0.03^b^
UFA	88.97 ± 0.11^e^	91.17 ± 0.14^b^	89.39 ± 0.10^d^	91.38 ± 0.06^a^	90.24 ± 0.05^c^
MUFA/PUFA	4.33 ± 0.73^b^	0.21 ± 0.00^d^	10.76 ± 0.04^a^	0.22 ± 0.00^d^	1.00 ± 0.01^c^
ω-6/ω-3 PUFA	0.85 ± 0.05^d^	4.94 ± 0.02^b^	19.20 ± 0.90^a^	0.20 ± 0.00^d^	4.00 ± 0.02^c^

Data are expressed as mean ± SEM (*n* = 3), and the values with different superscripts in the same row indicate statistically significant differences between groups as determined by one-way ANOVA at the level of *p* < 0.05. ND, not detected; ZanO, *Zanthoxylum bungeanum* seed oil; WalO, walnut oil; CamO, camellia seed oil; PerO, perilla seed oil; BLO, blended oil; SFA, saturated fatty acid; UFA, unsaturated fatty acid; MUFA, monounsaturated fatty acid; PUFA, polyunsaturated fatty acid.

### 3.2. Body and organ weights

**Figure 1:**
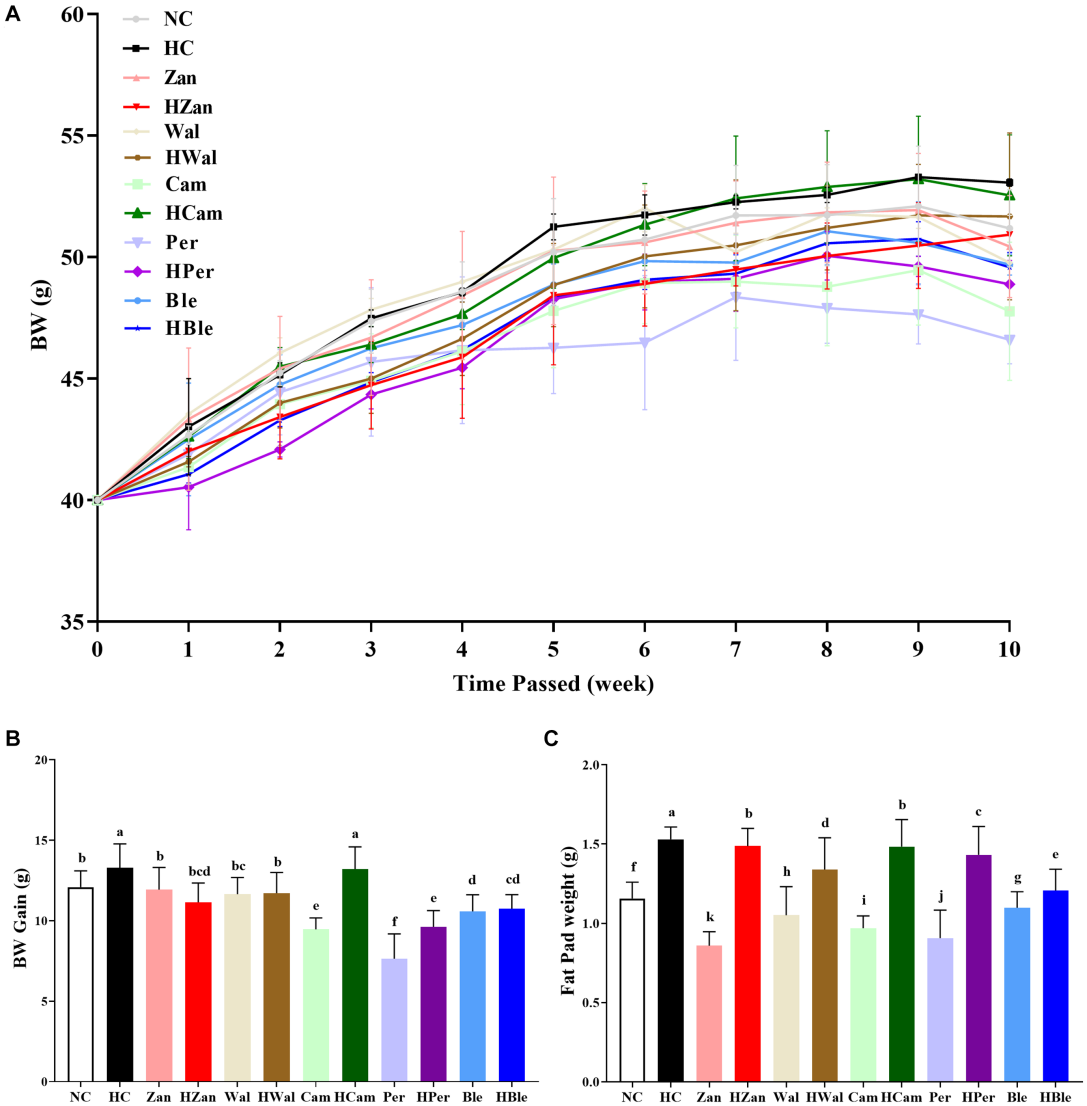
Effects of various oil supplements on the body and fat pad weights. **(A)** Body weight (BW), **(B)** body weight gain, and **(C)** epididymal fat pad weight. All data are expressed as mean ± SEM (*n* = 10 mice/group), and different superscripts indicate statistically significant differences between groups as determined by one-way ANOVA at the level of *p* < 0.05.

The evolution of the mice’s body weight (BW) as time passed is shown in Figure 1A. The BW of each mouse initiated from 40.0 g and gradually increased over time. The HC group had the highest final BW among all groups (53.06 ± 0.16 g), while the HBle group had a final BW of 49.58 ± 0.61 g. Similarly, the BW gain (Figure 1B) and epididymal fat pad weight (Figure 1C) of the HC group (13.29 ± 1.49 g and 1.53 ± 0.08 g, respectively) were higher than those of all other groups (*p* < 0.05). Comparatively, the corresponding values for the HBle group were 10.75 ± 0.86 g and 1.21 ± 0.13 g, respectively. Moreover, no statistically significant differences in spleen and liver weights (Supplementary Table 3) were observed between all the intervention groups and the HC group (*p* > 0.05).

### 3.3. Profiling of serum lipid and antioxidant activity in liver

**Figure 2:**
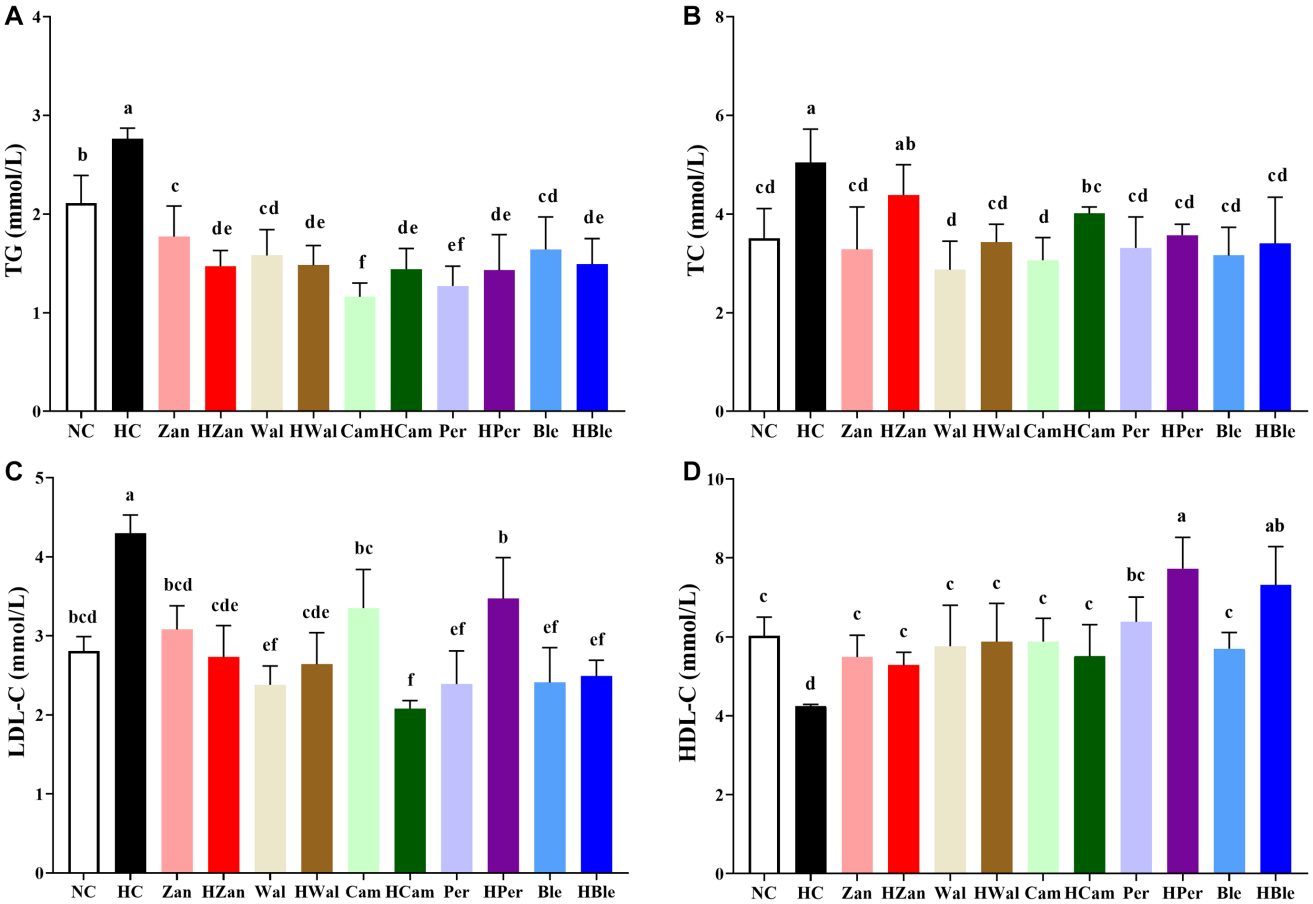
Effects of various oil supplements on the serum lipid levels. **(A)** Total triglyceride (TG), **(B)** total cholesterol (TC), **(C)** low-density lipoprotein cholesterol (LDL-C), and **(D)** high-density lipoprotein cholesterol (HDL-C). All data are expressed as mean ± SEM (*n* = 10 mice/group), and different superscripts indicate statistically significant differences between groups as determined by one-way ANOVA at the level of *p* < 0.05.

The HFD intervention significantly elevated the levels of TG (2.76 ± 0.11 mmol/L), TC (5.04 ± 0.68 mmol/L), and LDL-C (4.30 ± 0.23 mmol/L) in the HC group (Figures 2A–C). The HBle group showed significantly decreased levels (1.49 ± 0.36 mmol/L, 3.40 ± 0.94 mmol/L, and 2.49 ± 0.20 mmol/L, respectively). In contrast, the HDL-C level (Figure 2D) of the HC group (4.25 ± 0.04 mmol/L) was significantly lower than all other groups (*p* < 0.05), including the HBle group (7.31 ± 0.98 mmol/L).

**Figure 3:**
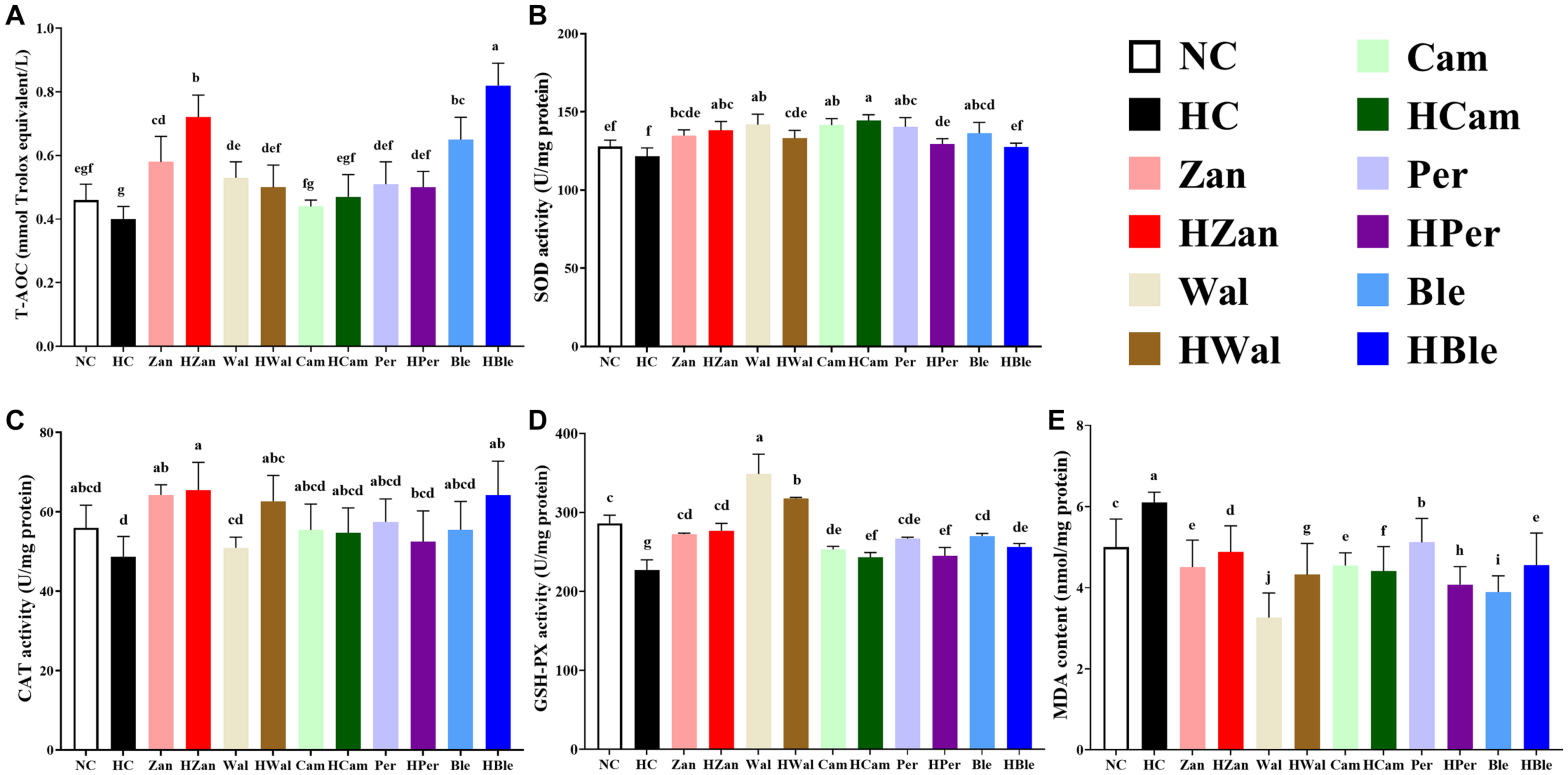
Effects of various oil supplements on the liver antioxidant activity levels. **(A)** Total antioxidant capacity (T-AOC), **(B)** superoxide dismutase (SOD), **(C)** catalase (CAT), **(D)** glutathione peroxidase (GSH-PX), and **(E)** malondialdehyde (MDA). All data are expressed as mean ± SEM (*n* = 10 mice/group), and different superscripts indicate statistically significant differences between groups as determined by one-way ANOVA at the level of *p* < 0.05.

Similar results were observed in the antioxidant activity levels of the homogenized mouse livers (Figure 3). The HFD intervention significantly reduced the levels of T-AOC (0.40 ± 0.03 mmol Trolox equivalent/L), SOD (121.54 ± 5.43 U/mg protein), CAT (48.62 ± 5.18 U/mg protein), and GSH-PX (226.93 ± 13.20 U/mg protein), while significantly increased the level of MDA (6.11 ± 0.25 nmol/mg protein) in the HC group (*p* < 0.05). In contrast, these values for the HBle group (0.82 ± 0.07 mmol Trolox equivalent/L for T-AOC, 64.20 ± 8.56 U/mg protein for CAT, 256.60 ± 4.14 U/mg protein for GSH-PX, and 4.55 ± 0.80 nmol/mg protein for MDA, respectively) were significantly reversed compared with the HC group (*p* < 0.05), except for the SOD level (*p* > 0.05). Notably, the HBle group peaked in the levels of T-AOC and CAT.

### 3.4. Histological analysis of liver

**Figure 4:**
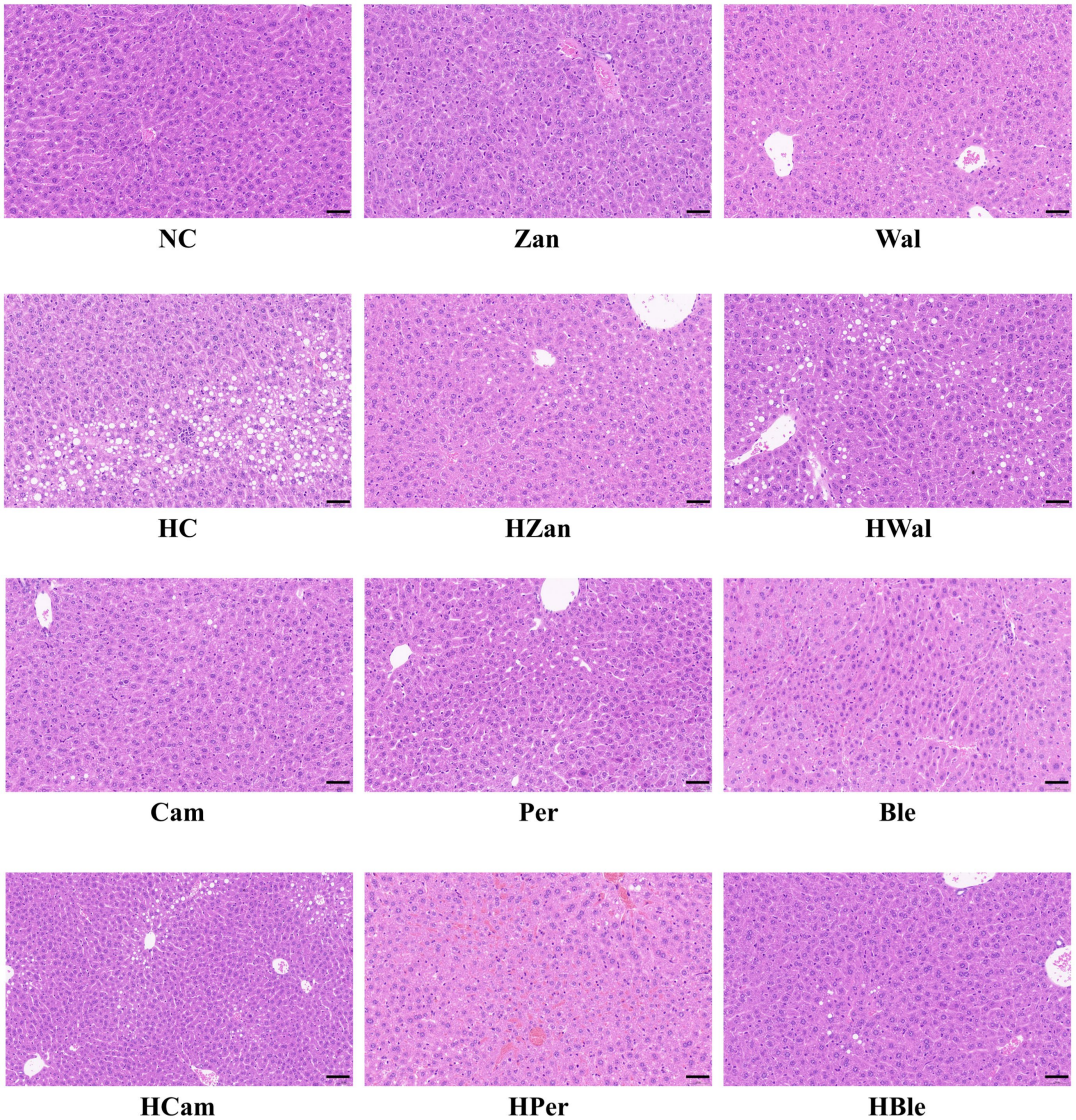
Effects of various oil supplements on the liver histopathology with H&E staining. Scale bar, 50 μm.

**Figure 5:**
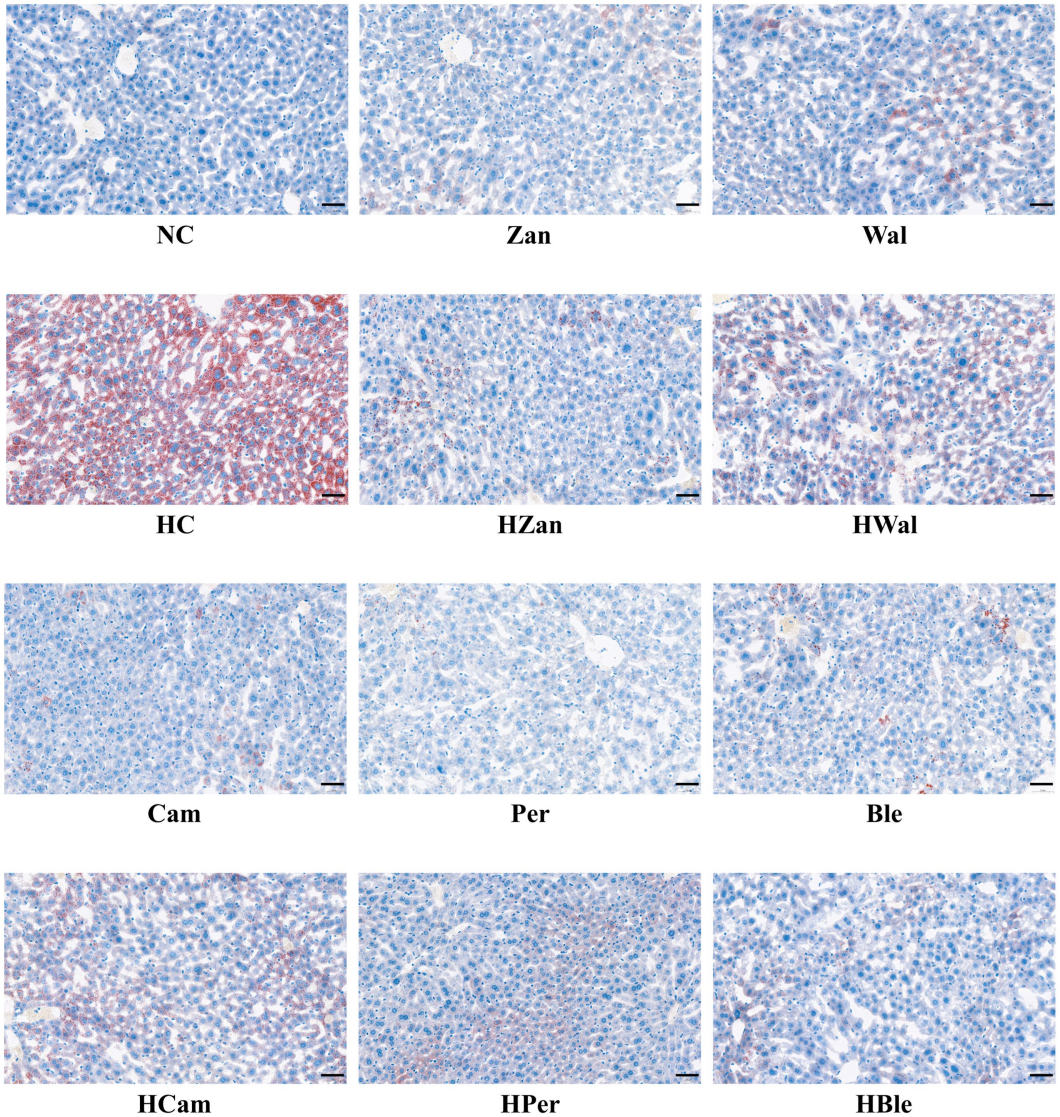
Effects of various oil supplements on the liver histopathology with Red Oil O staining. Scale bar, 50 μm.

The NC and other groups fed a chow diet exhibited no significant abnormalities. In contrast, the HC group showed typical fat accumulation of lipid droplets and hepatic steatosis, as observed in the H&E staining graphics (Figure 4). Furthermore, all the groups fed an HFD, including the HBle group, exhibited varying degrees of attenuation in fat accumulation and hepatic steatosis in liver cells compared with the HC group. This evidence was supported by the Oil Red O staining graphics (Figure 5).

### 3.5. Gut microbial analysis of cecal contents

**Figure 6:**
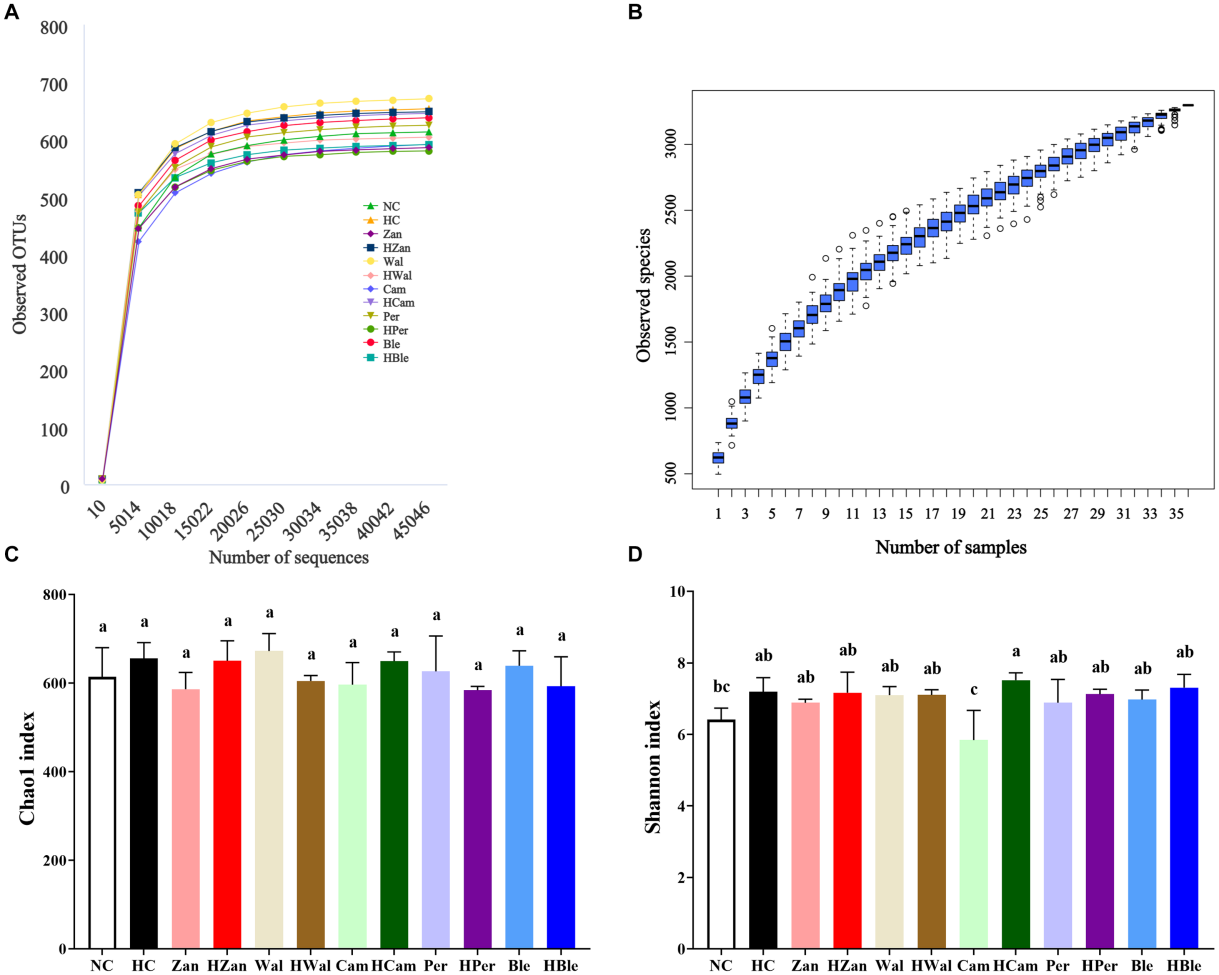
Effects of various oil supplements on the alpha diversity of gut microbiota. **(A)** Rarefaction curves (constructed with clusters with a sequence similarity value of 100% equivalent), **(B)** species accumulation boxplots, **(C)** Chao1 index, and **(D)** Shannon index. All data are expressed as mean ± SEM (*n* = 3 mice/group), and different superscripts indicate statistically significant differences between groups as determined by one-way ANOVA at the level of *p* < 0.05. OTUs, operational taxonomic units.

Sequencing the hypervariable regions of the bacterial 16S rDNA (V3–V4) generated a dataset of 2,171,716 effective tags from 36 samples (ranging from 52,688 to 76,872; average, 60,325). Figure 6A shows the rarefaction curves of the sequencing data that were flattened when the number of sequences reached 2 × 10^4^ reads, suggesting that the volume of sequencing data was gradually sufficient. More additional data would not significantly impact the alpha diversity index of the gut microbiota. Additionally, the species accumulation boxplot was constructed to investigate the sufficiency of the sample number, suggesting that 36 was sufficient for the gut microbiota analysis (Figure 6B). The Chao1 and Shannon indexes (Figures 6C,D), which could represent the diversity and abundance of microorganisms, did not show significantly differences (*p* > 0.05) among the different groups. The principal component analysis (PCA) revealed a separation between the HC and HBle groups, indicating the beta diversity of gut microbiota (Supplementary Figure 4).

**Figure 7:**
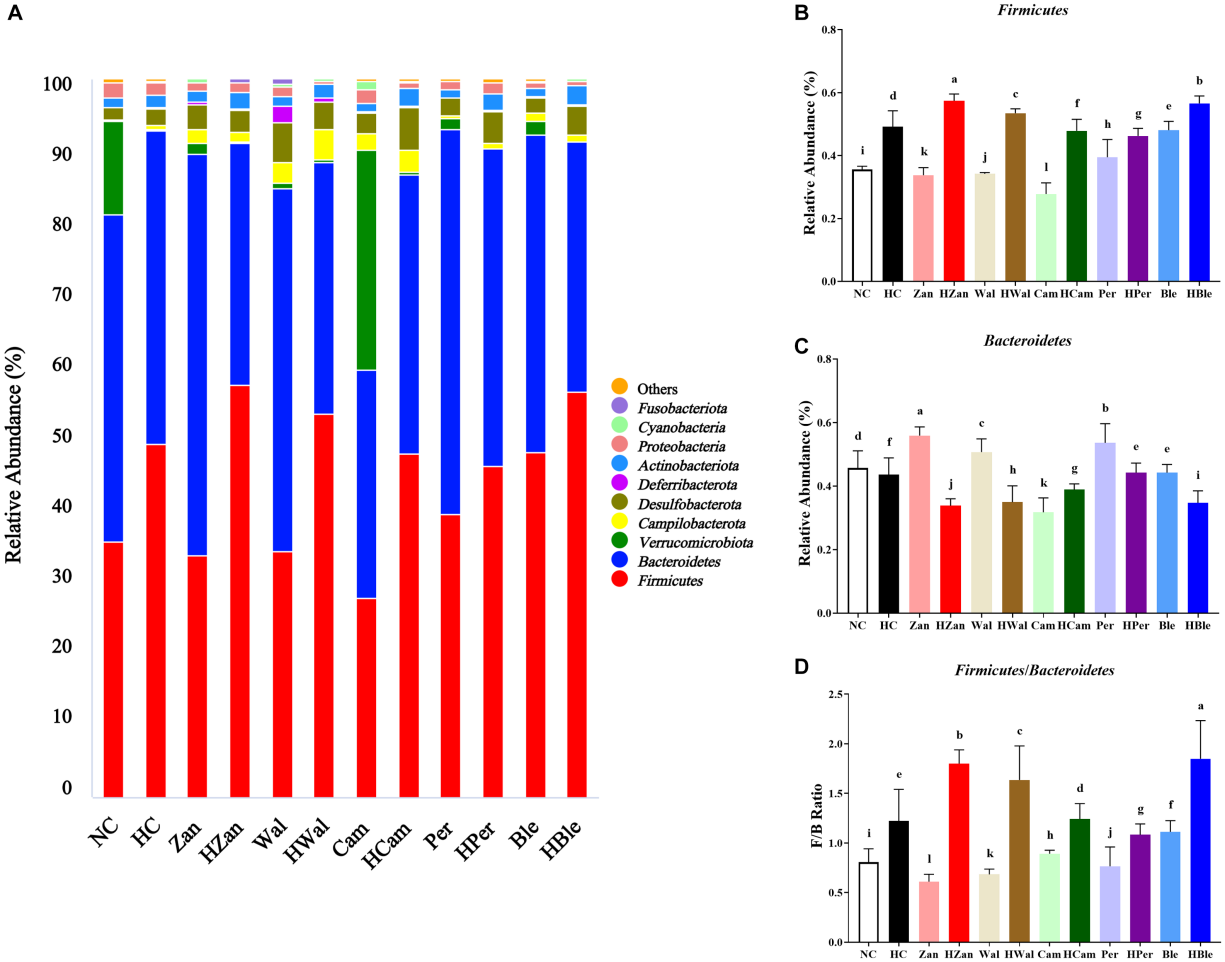
Effects of various oil supplements on the gut microbiota composition at the phylum level. **(A)** Relative abundance of the gut microbiota at the phylum level, and **(B–D)** relative abundance of the *Firmicutes*, *Bacteroidetes*, and the ratio of *Firmicutes/Bacteroidetes*, respectively. All data are expressed as mean ± SEM (*n* = 3 mice/group), and different superscripts indicate statistically significant differences between groups as determined by one-way ANOVA at the level of *p* < 0.05.

The relative abundance of microbial taxa was analyzed at the phylum and genus levels. At the phylum level (Figure 7A), the top 10 bacterial phyla identified were *Firmicutes, Bacteroidetes, Verrucomicrobiota, Campilobacterota, Desulfobacterota, Deferribacterota, Actinobacteriota, Proteobacteria, Cyanobacteria*, and *Fusobacteriota*. The abundance of *Firmicutes* in the mice’s cecal contents from the HC group was significantly higher than the NC group (*p* < 0.05; Figure 7B). Conversely, *Bacteroidetes* showed lower abundance in the HC group (*p* < 0.05; Figure 7C), resulting in a higher *Firmicutes/Bacteroidetes* ratio in the HC group (Figure 7D).

**Figure 8:**
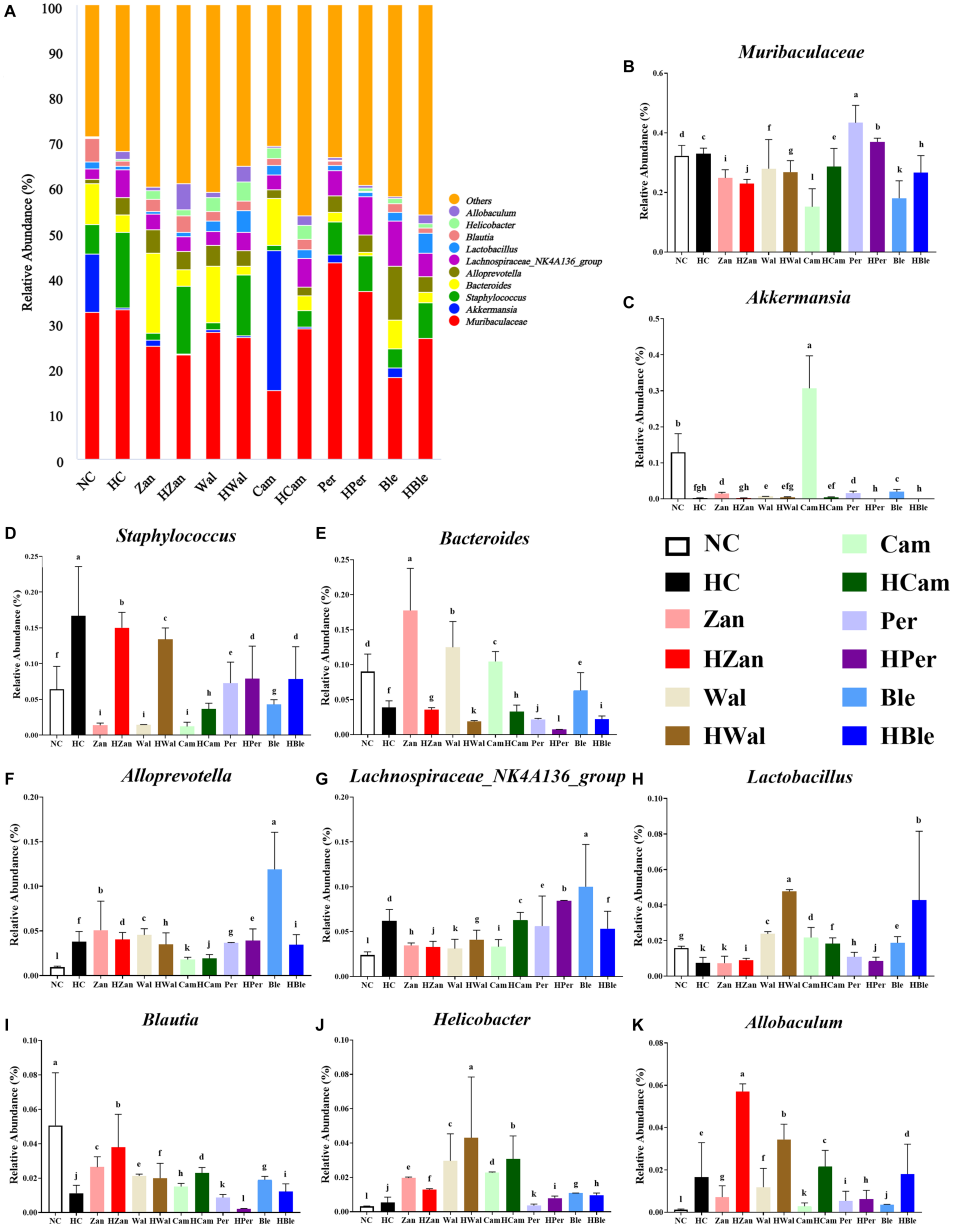
Effects of various oil supplements on the gut microbiota composition at the genus level. **(A)** Relative abundance of the gut microbiota at the genus level, and **(B–K)** relative abundance of the top 10 microbial species at the genus level. All data are expressed as mean ± SEM (*n* = 3 mice/group), and different superscripts indicate statistically significant differences between groups as determined by one-way ANOVA at the level of *p* < 0.05.

At the genus level (Figure 8A), the primary bacterial species were *Muribaculaceae, Akkermansia, Staphylococcus, Bacteroides, Alloprevotella, Lachnospiraceae_NK4A136_group, Lactobacillus, Blautia, Helicobacter*, and *Allobaculum*. Compared with the NC group, the HC group exhibited higher levels of *Muribaculaceae*, *Staphylococcus*, *Alloprevotella*, *Lachnospiraceae_NK4A136_group*, *Helicobacter*, and *Allobaculum* but lower levels of *Akkermansia*, *Bacteroides*, *Lactobacillus*, and *Blautia* (Figures 8B–K). The various oil supplements showed varying degrees of reversal of these changes. The HBle group showed increased levels of *Lactobacillus, Allobaculum*, and *Blautia* but reduced levels of *Staphylococcus* and *Bacteroides*, compared with the HC group (*p* < 0.05). Moreover, the Cam group exhibited a higher abundance of *Akkermansia* (*p* < 0.05); the abundance of *Staphylococcus* in all the intervention groups was lower than the HC group (*p* < 0.05); the HWal group exhibited higher levels of *Lactobacillus* (*p* < 0.05). Parallel results were observed in the Heatmap of the relative abundance of the altered microbial species in each group (Supplementary Figure 5).

### 3.6. Correlation analyses of specific gut microbiota with core host parameters

**Figure 9:**
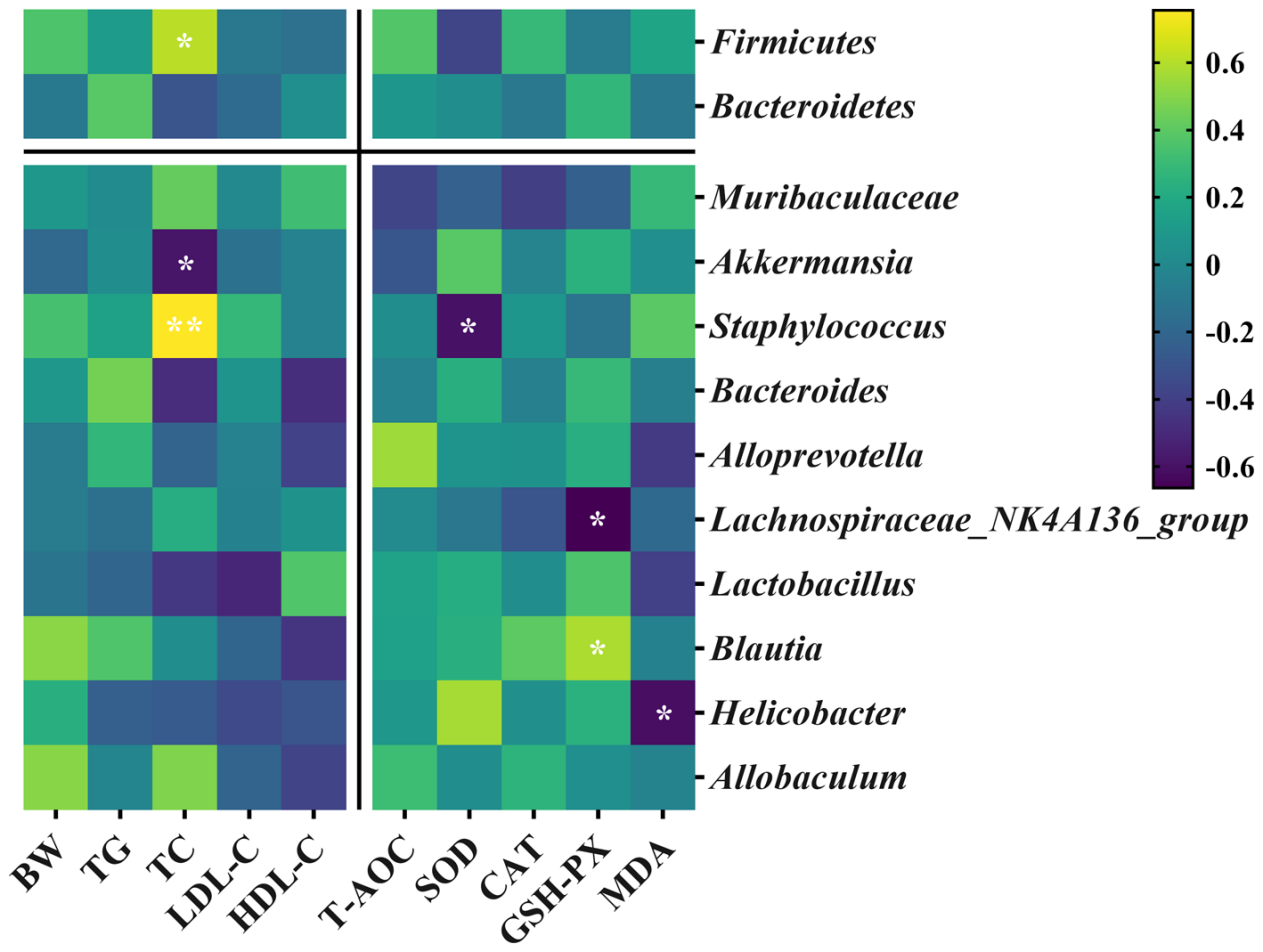
Heatmap of Spearman’s correlation analysis of specific gut microbiota with core host parameters of mice. Color intensity indicates the R-value of Spearman’s correlation (yellow indicates a positive correlation, while purple indicates a negative correlation). * and ** indicate statistically significant correlation of different degrees (*p* < 0.05 and *p* < 0.01, respectively).

Spearman’s correlation analysis explored the relationships between specific gut microbiota and core host parameters. As shown in Figure 9, *Firmicutes* was positively correlated with TC at the phylum level and negatively correlated with SOD. *Bacteroidetes* was negatively correlated with TC. At the genus level, *Muribaculaceae* was negatively correlated with T-AOC and CAT; *Akkermansia* was positively correlated with SOD but negatively correlated with TC; *Staphylococcus* was positively correlated with TC yet negatively correlated with SOD; *Bacteroides* was negatively correlated with TC and HDL-C; *Alloprevotella* was positively correlated with T-AOC and negatively correlated with HDL-C and MDA; *Lachnospiraceae_NK4A136_group* was negatively correlated with CAT and GSH-PX; *Lactobacillus* was negatively correlated with TC, LDL-C, and MDA, but it was positively correlated with HDL-C and GSH-PX; *Blautia* was positively correlated with GSH-PX but negatively correlated with HDL-C; *Helicobacter* was positively correlated with SOD yet negatively correlated with MDA; *Alloprevotella* was positively correlated with BW and TC, and negatively correlated with HDL-C.

## 4. Discussion

The intake of dietary fats, which are classified into SFAs, MUFAs, and PUFAs, is strongly linked to host metabolism (5). Prolonged consumption of an SFA-rich HFD promotes the excessive deposition of lipids in the liver, leading to overnutrition, inflammation, and hepatic steatosis (31). UFAs, abundant in vegetable oils, have potential preventive effects against various metabolic disorders, including cardiovascular disease (32) and obesity (33, 34). Previous studies have revealed the intrinsic mechanism of lipid regulation involving UFAs. The activation of PPAR could enhance the β-oxidation of fatty acids, thus reducing the synthesis of very low-density lipoprotein (VLDL) in the liver (35, 36). The Mediterranean dietary pattern, characterized by a significant proportion of MUFAs in olive oil (37), has been associated with a lower incidence of chronic diseases among its residents (38). This incidence is three times lower than that observed in the United States, despite similar levels of total fat intake (32). In addition, essential PUFAs serve as precursors for various biochemical molecules, including leukotrienes, prostaglandins, thromboxanes, and phospholipids, which exert metabolic bioactivities via gene transcriptional or epigenetic processes (39–41). The principal ω-3 PUFAs obtained from dietary sources are eicosapentaenoic acid (EPA), docosahexaenoic acid (DHA), and alpha-linolenic acid (ALA). On the other hand, linoleic acid (LA) and arachidonic acid (ARA) are the major ω-6 PUFAs derived from food (42). PUFAs can be metabolized to generate bioactive metabolites, such as eicosanoids and docosanoids, which have the potential to activate G protein-coupled receptors (GPCRs) and regulate inflammatory processes (43). ARA metabolites are crucial for a multitude of biological processes (e.g., lipid and protein metabolism regulation, leukocyte and platelet function activation). DHA metabolites (e.g., protectins, maresins, and resolvins) could act as biochemical regulators in inhibiting the genesis of pro-inflammatory cytokines, thereby suppressing neutrophil migration and enhancing phagocytosis (34, 44, 45). Furthermore, a previous study has indicated that as the degree of oil unsaturation increases, such as PUFAs do, there is a corresponding decrease in liver TAG levels (46).

Nevertheless, certain metabolic by-products of PUFAs, particularly prostaglandins and leukotrienes derived from ω-6 PUFAs, exhibit pro-inflammatory properties during the inflammatory cascade (47). Moreover, ω-3 and ω-6 PUFAs compete for the enzymes responsible for elongation and desaturation, depending on their respective levels during bioconversion (48). The nervous system, particularly the brain, contains the highest concentration of lipids among all human organs, with lipids comprising 60% of its dry weight, of which 35% are PUFAs (14, 49). Therefore, maintaining a balanced ratio of PUFA is more significant than the overall quantity of PUFAs. Excessive ω-6 PUFAs may lead to a pro-inflammatory response by the corresponding prostaglandins or leukotrienes derivates (47). Imbalances in UFAs could contribute to the development of specific diseases and increase the risk of death (12). Notably, an elevated ω-6/3 PUFA ratio in maternal diets could even conduce to the inter-generational cycle of obesity (50). Studies have indicated the relevance between chronic metabolic disorders and increased ω-6/3 PUFA ratio in diets (13). The BLO with a modified ω-6/ω-3 PUFA ratio has shown improved nutritional and functional properties, benefiting host metabolism via regulating the lipid metabolic signaling pathways and pro-inflammatory cytokines (15, 46, 51–53).

Currently, medical or behavioral interventions are insufficient for long-term weight loss (1), and could lead to side effects or high recurrence rates (54). Meanwhile, dietary intervention is the principal strategy to control obesity owing to its safety and social acceptability (2). The quality of the ingested dietary oil is crucial for maintaining health, and vegetable oil is a promising dietary supplement for its wholesome UFAs and other bioactive components (7–9). Many dietary guidelines suggest substituting SFAs with UFAs and recommend a balanced ratio of UFAs (20, 21, 55). The UFA-rich woody edible oils contain unique nutrients (e.g., antioxidant peptides, phytosterols, and polyphenols) that benefit host health, compared with common edible oils (8). BLO is now widely accepted owing to no single oil with optimal nutritional properties (16–19). However, most published studies on the BLO involve olive or fish oil (22, 24) that are not easily accessible in most regions. The woody oil plants are abundant in Asia and widely spread in Guizhou Province, southwest China (9, 28). Based on the ZanO, WalO, CamO, and PerO, we developed the BLO, a balanced UFA supplement, in which the ratios of MUFAs/PUFAs and ω-6/ω-3 PUFAs were 1:1 and 4:1, respectively. Due to the correlations between UFAs and lipid regulation (34–36, 45), we conducted animal experiments to assess the effects of the BLO on HDF-induced mice.

Long-term consumption of HFD is the most common cause of hyperlipidemia, leading to aberrant lipid metabolism in the liver that could develop into hepatic steatosis and non-alcoholic fatty liver (31, 56). Hyperlipidemia generally accompanies increased TG, TC, LDL-C, and decreased HDL-C (46). In this study, the KM mice fed an HFD for 10 weeks developed hyperlipidemia with typical changes (Figure 2) and hepatic steatosis with increased fat deposition in cells (Figures 4, 5). Compared with the HFD blank control group (HC group), the mice fed with the BLO supplement (HBle group) exhibited decreased BW (Figures 1A, B), epididymal fat pad weight (Figure 1C) and reduced levels of TG, TC, and LDL-C (Figures 2A–C) but increased levels of HDL-C (Figure 2D). Meanwhile, the histological graphs of H&E and Red Oil O staining showed that the BLO supplement effectively attenuated the fat accumulation in the liver cells (Figures 4, 5). TAG is the primary accumulative lipid component in the liver, which is regulated by the synthetic and oxidative processes (57). Previous studies have indicated that the regulatory factors involving fatty acid oxidation and adipogenesis are influenced by dietary fat composition and that PUFAs are the preferred ligands for the β-oxidation of fatty acid (39, 52, 58). LDL-C and HDL-C are responsible for dissolving and transporting cholesterol and TAG, and TAGs in lipoprotein particles could be hydrolyzed into fatty acids (52). Furthermore, LDL-C could be oxidized and adhere to the arterial wall, increasing atherosclerosis risk (59). In contrast, HDL-C could prevent LDL-C from oxidizing and take part in reverse cholesterol transport from blood to the liver (60). These bioactive molecules are vital for lipid metabolism. Our findings concur with previously published studies involving UFAs (22–24, 39, 61) and indicate that the BLO supplement effectively ameliorate lipid metabolism and fat accumulation in the liver cells of KM mice.

HFD could influence the oxidant and antioxidant balance in the human body (62). ROS, a by-product generated during almost all metabolic processes, is enhanced with HDF ingestion (62). ROS participates in various life processes (e.g., mitochondrial activity, lipogenesis, and adipogenesis) and plays significant roles in the pathogenesis of obesity and non-alcoholic fatty liver disease (63). For instance, lipid peroxides are generated under ROS inducement and could destroy cell membranes, releasing intracellular aldehyde compounds, such as malondialdehyde (MDA) (64). Antioxidant enzymes, such as superoxide dismutase (SOD), catalase (CAT), and glutathione peroxidase (GSH-PX), could help reduce the free radical cytotoxicity from ROS (65). Thus, minimizing MDA formation and maximizing the antioxidative enzyme activities are practical for obesity prevention. In this study, the HBle group exhibited lower levels of MDA and higher levels of CAT, GSH-PX, and total antioxidant capacity (T-AOC) than the HC group (Figure 3). In addition, while all groups with various oil supplements showed improvements in serum lipid levels and antioxidant activities to varying degrees, consistent with previous studies (15, 66), the group with the BLO supplement performed the best in terms of TC, LDL-C, HDL-C, and T-AOC levels. The findings may be associated with the modified ratios of UFAs.

The ratio of ω-6/ω-3 PUFA plays a crucial role in maintaining the homeostasis of gut microbiota and promoting intestinal health (6). Previous studies have demonstrated the correlations between the richness and diversity of gut microbiota and lipid metabolism, suggesting that gut microbiota could serve as the diagnostic and therapeutic biomarkers of hyperlipidemia (6, 67). In this study, various oil supplements did not alter the alpha diversity of gut microbiota (indicated by the Chao1 and Shannon indexes; Figure 6) but influenced the gut microbiota compositions at the phylum and genus levels (Figures 7, 8). *Firmicutes* and *Bacteroidetes* are the dominant bacterial species at the phylum level. Previous studies have suggested that an increased ratio of *Firmicutes/Bacteroidetes* is usually accompanied by obesity (68), although contrasting results have also been reported (69). However, recent research has indicated that altering gut microbiota composition at lower taxonomic levels may have more significant health benefits (70). Our results showed that the BLO supplement increased the abundance of *Allobaculum*, *Lactobacillus*, and *Blautia,* while reducing the abundance of *Staphylococcus* in mice fed an HFD compared with the HC group (Figure 8). The heatmap of Spearman’s correlation analysis indicated that these gut microbial species were associated with ameliorated serum lipid levels and antioxidative activities (Figure 9). *Allobaculum* could benefit butyrate production, which is one of the three primary short-chain fatty acids (SCFAs) possessing anti-obesity and anti-inflammatory properties. It can up-regulate the expression of angiopoietin-like 4 (ANGPTL4), PPAR-γ, and HDL-C, resulting in the enhanced lipid metabolism (71–75). Hence, SCFA production may be a potential mechanism by which the BLO supplement regulates lipid metabolism (Figure 8K). A previous study has shown that butyrate promotes the growth of *Lactobacillus* in the colon (76), and *Lactobacillus*, in turn, regulates gut lipid metabolism by competing for fatty acid with the host (77). Moreover, the organic acids produced by *Lactobacillus* act as ideal electron donors, scavenging the free radicals such as hydroxyl and superoxide (78, 79). Spearman’s correlation analysis revealed positive correlations between *Lactobacillus* abundance, enriched by the BLO supplement (Figure 8H), and levels of HDL-C, T-AOC, SOD, CAT, and GSH-PX, as well as negative correlations with TG, TC, LDL-C, and MDA (Figure 9). In addition, the enrichment of *Blautia* by the BLO supplement (Figure 8I) has been reported to be associated with ameliorated fat accumulation (80) and intestinal barrier integrity (81). Spearman’s correlation analysis indicated positive correlations between *Blautia* abundance with GSH-PX levels (Figure 9). On the other hand, the facultative *Staphylococcus*, inhibited by the BLO supplement (Figure 8D), is commonly elevated in hyperlipidemia and overweight individuals (29, 82) and is considered as a potential pathogen (83). Spearman’s correlation analysis presented a significantly positive correlation between *Staphylococcus* abundance and TC levels (*p* < 0.01), as well as a negative correlation with SOD (*p* < 0.05; Figure 9). These findings suggest that the BLO supplement might regulate lipid metabolism and antioxidant activity by re-establishing the gut microbiota. Remarkably, we found that the CamO supplement could tremendously increase the relative abundance of *Akkermansia* in the mice fed a chow diet (Figure 8C). *Akkermansia* is now a research hotspot as a potential probiotic, which produces SCFAs and branched-chain fatty acids (BCFAs) associated with host metabolism, inflammation, and gut microbiota (73, 84, 85). Moreover, the outer membrane protein of *Akkermansia*, which is thermally stable under pasteurization, could interact with Toll-like receptor 2 (TLR-2) and benefit the gut barrier (86). This finding provides hints for our future research.

The upsurge in total lipid consumption has dramatically impacted human health in recent years. The significance of dietary intervention is emphasized in various contexts. Dietary supplements (e.g., probiotics and functional foods) could exhibit reliable influences on metabolism regulation (67, 77). Woody oil plants are widely spread worldwide, and woody edible oil has gained increasing importance owing to its desirable nutritional qualities (8). In this study, the BLO, a balanced UFA supplement, was achieved based on woody edible oils and could exhibit specific metabolic regulatory properties in KM mice. However, in addition to MUFA and PUFA, the BLO also contains trace amounts of other fatty acids, such as palmitic acid (C16:0), stearic acid (C18:0), and arachidic acid (C20:0). These fatty acids, along with other bioactive substances present in trace concentrations, may potentially affect the gut microbiota and lipid metabolism in mice. Additionally, it is important to note that the molecular mechanism remains unclear. The secondary metabolites of gut microbiota, such as SCFAs, play a significant role in metabolic improvement. These SCFAs can interact with molecules such as ANGPTL4 and PPAR-γ. PUFA, which acts as a common ligand for PPAR, also participates in the process of lipid metabolism. In this study, it is possible that changes in these molecules contributed to metabolic improvement, and further intensive research is necessary to verify the nutritional functions of the BLO.

## 5. Conclusion

In this study, the BLO, a balanced UFA supplement, achieved an ideal ratio of UFAs and was administrated to HFD-induced KM mice as a dietary supplement. We systematically investigated its influences on lipid metabolism, antioxidant activity, and gut microbiota. Our results showed that the BLO supplement reduced the body and epididymal fat pad weights, decreased liver fat accumulation, ameliorated serum lipid levels, and facilitated liver antioxidant activities. Moreover, the BLO supplement increased the abundance of *Lactobacillus, Allobaculum*, and *Blautia* and reduced the abundance of *Staphylococcus*, which might be associated with improved lipid metabolism and antioxidant activities. The findings indicated that the BLO could be a practical oil supplement for preventing hyperlipidemia and obesity.

## Data availability statement

The 16S sequencing datasets generated in this study are available in online repositories (NCBI SRA) under the accession number PRJNA954531. For further inquiries regarding the datasets, please contact the corresponding authors.

## Ethics statement

The animal study was reviewed and approved by Laboratory Animal Ethics Committee of Guizhou University.

## Author contributions

XC: software, formal analysis, investigation, data curation, writing—original draft, and writing—review and editing. JR: software, formal analysis, investigation, and data curation. MM: investigation, resources, and visualization. YZ: validation and funding acquisition. YL: data curation and visualization. LQ: validation, conceptualization, methodology, resources, project administration, and funding acquisition. SM: conceptualization, methodology, and supervision. All authors contributed to the article and approved the submitted version.

## Funding

This study was funded by Guizhou Science and Technology Program [Qian Ke He Zhi Cheng (2022) zhongdian 005], Research Center of Engineering Technology of Guizhou Province [Qian Ke He Ping Tai Ren Cai (2020) 2105], National Natural Science Foundation of China [Grant No. 32001352], and National Key Research and Development Program of China (No. 2022YFD1100305).

## Conflict of interest

The authors declare that the research was conducted in the absence of any commercial or financial relationships that could be construed as a potential conflict of interest.

## Publisher’s note

All claims expressed in this article are solely those of the authors and do not necessarily represent those of their affiliated organizations, or those of the publisher, the editors and the reviewers. Any product that may be evaluated in this article, or claim that may be made by its manufacturer, is not guaranteed or endorsed by the publisher.
