# Supplementary material for: The balanced unsaturated fatty acid supplement constituted by woody edible oils improved lipid metabolism and gut microbiota in high-fat diet mice
Source: Front Nutr. 2023 Jul 20;10:1203932. doi: 10.3389/fnut.2023.1203932 (PMC10399753; doi:10.3389/fnut.2023.1203932)
Supplement: Supplementary file 1 [file Data_Sheet_1.PDF]

Supplementary Material

The Balanced Unsaturated Fatty Acid Supplement Constituted by Woody Edible Oils Improved Lipid Metabolism and Gut Microbiota in High-Fat Diet Mice

Xi Chen[1,3], Jingqi Ran[2], Mazhar Muhammad[1,2], Yong Zhu[2], Yichen Lin[4], Likang Qin[1,2,*], Song Miao[4,*]

[1] Key Laboratory of Plant Resource Conservation and Germplasm Innovation in Mountainous Region (Ministry of Education), College of Life Sciences/Institute of Agro-Bioengineering, Guizhou University, Guiyang, China

[2] School of Liquor and Food Engineering, Guizhou University, Guiyang, China

[3] Department of Laboratory Medicine, Affiliated Jinyang Hospital of Guizhou Medical University, Guiyang, China

[4] Teagasc Food Research Centre, Moorepark, Fermoy, Co.Cork, Ireland

[*] **Correspondence**: Likang Qin, lkqin@gzu.edu.cn

Song Miao, songmiao@teagasc.ie

```
Command Window
>> format long
B=[0.7206,0.1595,0.8179,0.1651;0.0774,0.6255,0.0722,0.1258;0.0917,0.1267,0.0038,0.6229]
E=B(1,:);F=B(2,:);G=B(3,:);
for i=4:6
M=F-i*G;
N=E-(F+G);
A=[M;N];
c=[3,19.8,17,19.8];
b=[0;0];
Aeq=[1,1,1,1];
beq=[1];
vlb=[0.15,0,0.23,0.001];
[x,fval]=linprog(c,A,b,Aeq,beq,vlb,[])
m=B*x
h=sum(x)
n=m(1,1)/(m(2,1)+m(3,1))
o=m(2,1)/m(3,1)
end
```

Supplementary Figure 1. Codes for oil blending (MATLAB software).

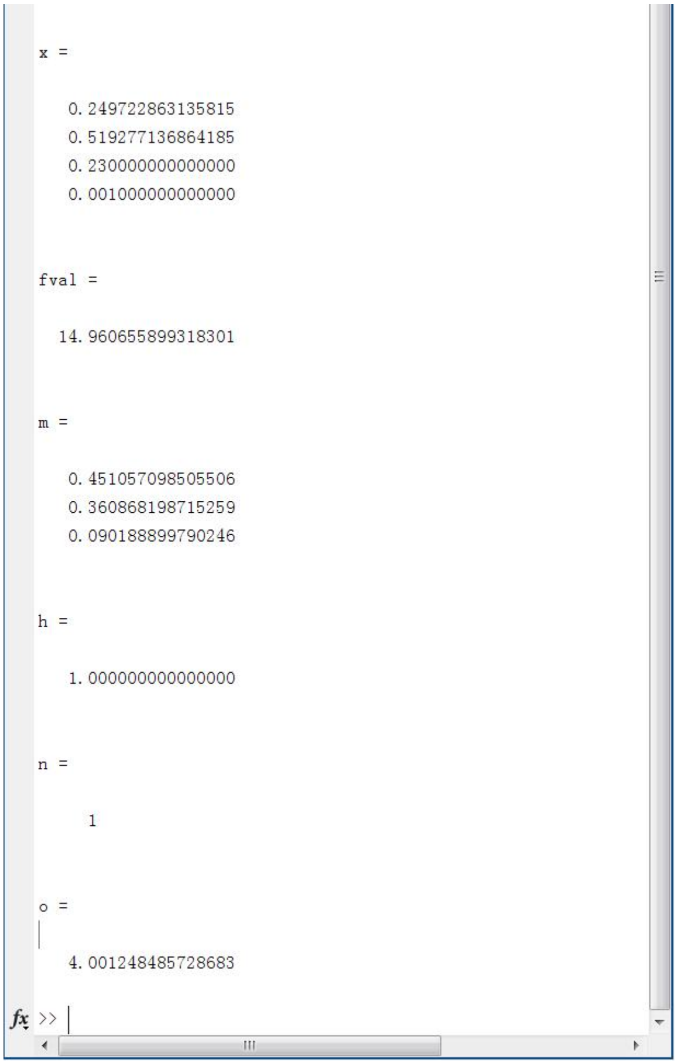

Supplementary Figure 2. Computational results for oil blending (MATLAB software).

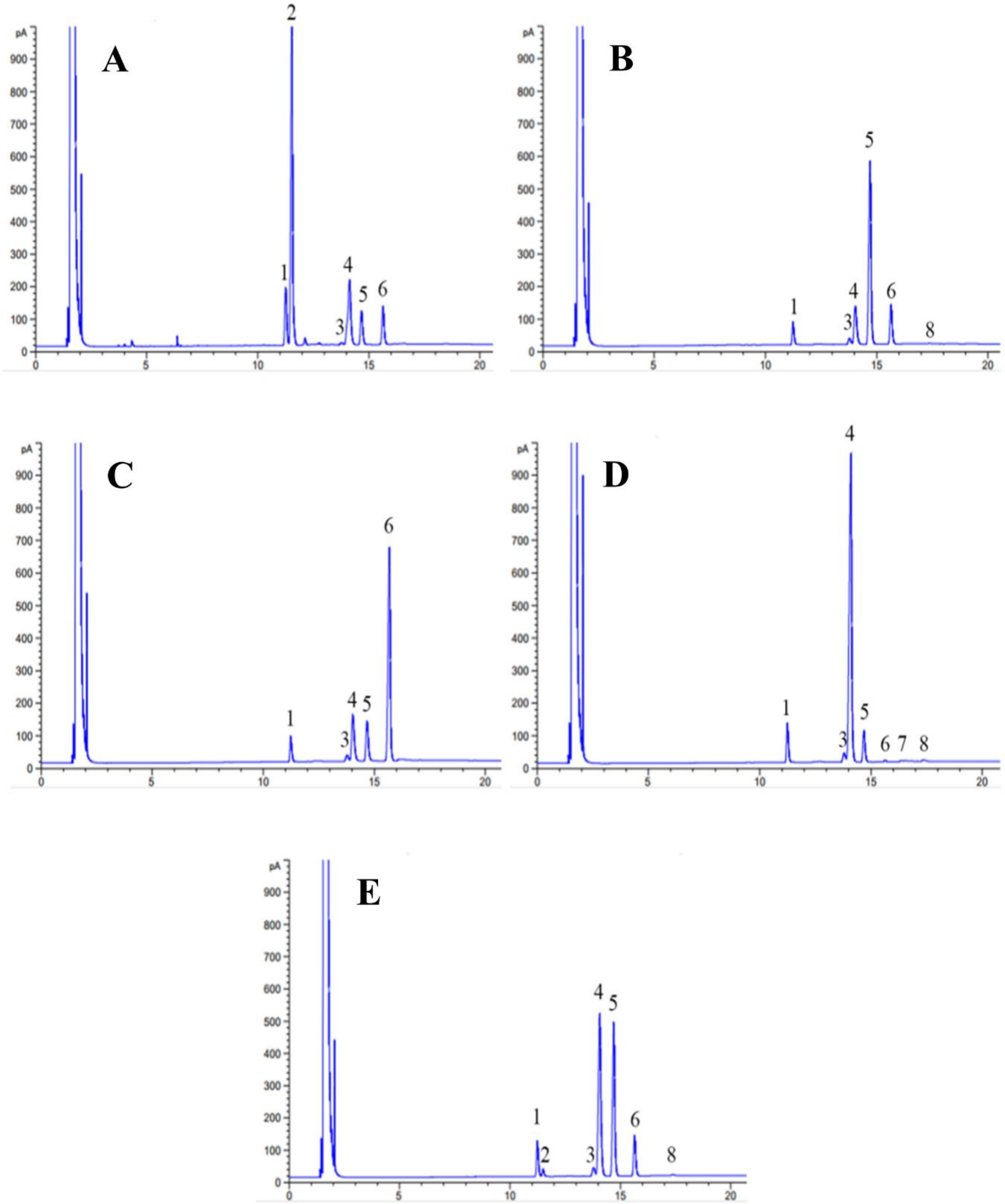

Supplementary Figure 3. Gas chromatography chromatograms of *Zanthoxylum bungeanum* seed oil **(A)**, walnut oil **(B)**, perilla seed oil **(C)**, camellia seed oil **(D)**, and blended oil **(E)**. The superscript numbers of 1 to 8 represent the peaks of palmitic acid (C16:0), palmitoleic acid (C16:1), stearic acid (C18:0), oleic acid (C18:1), linoleic acid (C18:2), α-linolenic acid (C18:3), arachidic acid (C20:0), and eicosenoic acid (C20:1), respectively.

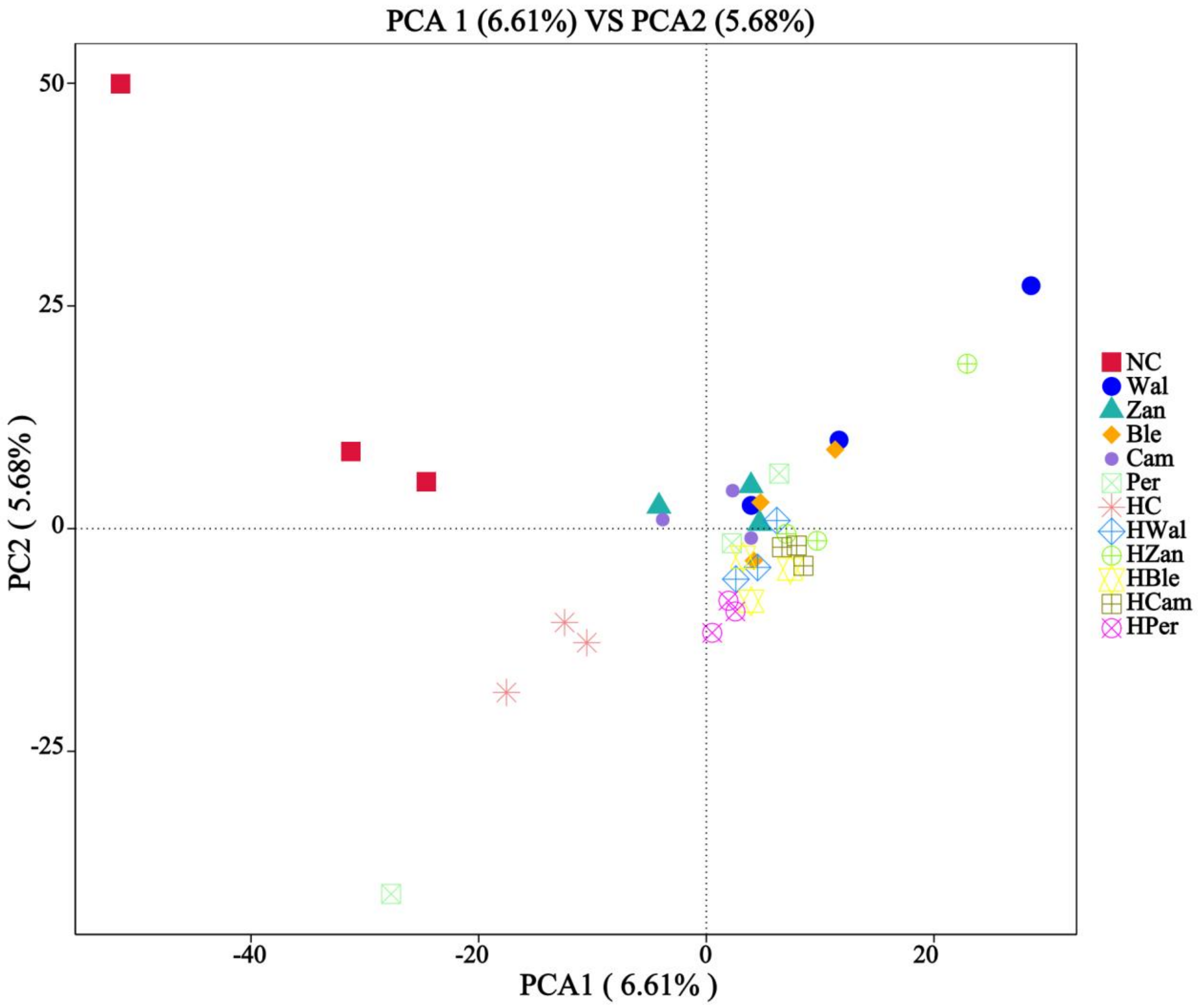

Supplementary Figure 4. Principal component analysis (PCA) of gut microbiota communities (n = 3/group).

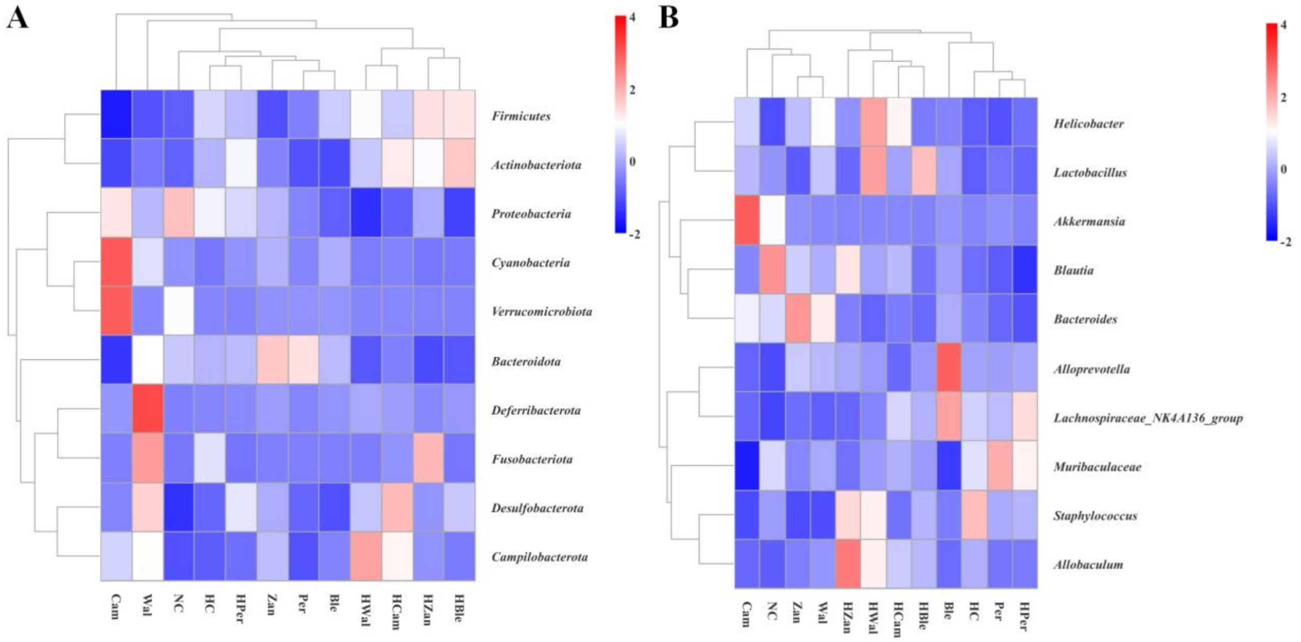

Supplementary Figure 5. Heatmaps of the gut microbiota in each group of mice at the phylum level **(A)** and the genus level **(B)**.

Supplementary Table 1. Composition of chow diet

Nutrients	Main ingredient
proteins	soybean meal, fish meal
fats	vegetable oil
fibers	bran
carbohydrates	corn, wheat middling
vitamins	VitA、VitD、VitE、VitB1、VitB2、VitB6、pantothenic acid
minerals	calcium hydrogen phosphate, rock powder, iron, copper, manganese, zinc, etc.

Supplementary Table 2. Nutrient composition guarantee values of chow diet

Nutrients (%)	Ingredient
moisture	≤ 10.0
crude protein	≥ 18.0
crude fat	≥ 4.0
crude fiber	≤ 5.0
crude ash	≤ 8.0
calcium	1.0 ~ 1.8
phosphorus	0.6 ~ 1.2

Supplementary Table 3. Organ weights

	Spleen weight (g)	Liver weight (g)
NC	0.14 ± 0.05[abc]	1.90 ± 0.15[ab]
HC	0.15 ± 0.04[ab]	1.94 ± 0.18[ab]
Zan	0.14 ± 0.05[abc]	1.91 ± 0.27[ab]
HZan	0.13 ± 0.04[bc]	1.77 ± 0.25[ab]
Wal	0.16 ± 0.04[ab]	1.95 ± 0.14[a]
HWal	0.15 ± 0.04[abc]	1.74 ± 0.13[ab]
Cam	0.12 ± 0.03[abc]	1.75 ± 0.17[ab]
HCam	0.11 ± 0.04[bc]	1.76 ± 0.22[ab]
Per	0.13 ± 0.05[abc]	1.64 ± 0.24[ab]
HPer	0.11 ± 0.03[c]	1.54 ± 0.12[b]
Ble	0.15 ± 0.04[abc]	1.87 ± 0.15[ab]
HBle	0.15 ± 0.04[abc]	1.75 ± 0.16[ab]

*Data are expressed as mean ± SEM (n = 10 mice/group), and the values with different superscripts in the same row indicate statistically significant differences between groups as determined by one-way ANOVA followed by Tukey's multiple comparison test. *$p < 0.05$.
